# Reversal of type 2 diabetes mellitus through integrated Ayurveda dietary protocol – A case report

**DOI:** 10.1016/j.jaim.2024.100946

**Published:** 2024-07-23

**Authors:** Suketha Kumari, Basavaraj R. Tubaki, Rekha S. Patil, S.D. Laxmikant

**Affiliations:** a Department of Kayachikitsa, KAHER's Shri B M Kankanawadi Ayurveda Mahavidyalaya, Shahapur, Belagavi, Karnataka, India, 590003; b Department of Medicine, KAHER's Jawaharlal Nehru Medical College, Nehru Nagar, Belagavi, Karnataka, India, 590003; c Department of Shalyatantra, KAHER's Shri B M Kankanawadi Ayurveda Mahavidyalaya, Shahapur, Belagavi, Karnataka, India, 590003

## Abstract

Type 2 Diabetes Mellitus (T2DM) is a chronic metabolic disorder of hyperglycemia has close link with obesity and insulin resistance. Standard approaches in T2DM management are lifestyle management and Pharmacotherapy. Dietary management in T2DM was found to be safe and effective. In India, studies on reversal of T2DM through diet were less found. Presenting a case of female aged 31 years with T2DM (HbA1c-13.5) was successfully reversed diabetes with dietary principles of Indian traditional medicine (Ayurveda diet) and therapeutic yoga practices. Pre and post assessment of Glycaemic parameters (HbA1C, FBS, and PPBS), Lipid profiles, Insulin resistance parameter (HOMA IR) were done. Major outcome of this case is greater reduction of HbA1c from inadequate control to near normal. Marked changes observed in weight, BMI and Waist circumference. A change in Homa IR indicates improvement in insulin resistance. Ayurveda dietary management training include Therapeutic fasting [Two meal method], meals include nutrient dense wholesome food, Indian millet based diet, herbal recipes [therapeutic food/herbal drinks, smoothies and plant juices] and eat only when hungry method. Yoga include complete sequel of body loosening practices, surya namaskara, yogic postures and breathing and meditative techniques practiced for specified period [60 min].This case showed effectiveness of Ayurveda diet and Yoga practices in reversing the insulin resistance, help to maintain the glycemic parameters .In addition, patient was able to loose body weight, waist circumference & serum triglyceride levels. Patient can able to withstand her diabetic profile in normal without any pharmacotherapy intervention.

## 1 Introduction

Type 2 Diabetes Mellitus (T2DM) is a chronic metabolic disorder associated with hyperglycemia, often linked to obesity and insulin resistance [1]. Approximately 50% of T2DM patients fail to achieve adequate glycemic control [Glycated hemoglobin (HbA1c < 7%)] [2] which is often attributed to poor adherence to oral hypoglycemic agents [3] primarily due to their adverse events [4]. Also, adherence to medication alone may not prevent the disease progression [5]. The standard approach to T2DM involves lifestyle management and pharmacotherapy. The American Diabetic Association (ADA) recommends various diets like Mediterranean, DASH, low-carbohydrate, and vegetarian diets in the US and Europe [6]. However, studies on dietary management of T2DM in India are limited [7]. Medical Nutrition Therapy (MNT) offers an evidence-based approach [8], but its implementation faces challenges. Globally, lifestyle interventions are not universally practiced [9], and in India, less than 5–10% of physicians recommend dietary intervention as the first-line management for T2DM [10] (see Table No.1, Table No.2).

**Table No.1:** Timeline of treatment modalities and measured health parameters at each follow up

Date of visit	Clinical events	Treatment at the time of visit	FBS (mg/dl)	PPBS (mg/dl)	HBA1C (%)	Weight (KG)	Waist circumference (cm)	BMI
Jan 2019	Dry palate, perspiration, Polyuria	First diagnosis	212	284	8.9	78	–	30.1
March 2019	Previous symptoms reduced, complaints of bloating and often hypoglycemic episodes	Metformin 1000mg two times a day	134	168	7.8	75	–	28.9
December 2019	Polyuria and polyphagia symptoms	Not on medication(self-dropped)	192	298	9.2	76	–	29.3
February 2021	Previous diabetic symptoms reduced. Had complaints of bloating and frequent unsatisfactory bowel movements	On metformin 1000mg twice a day(restarted medication on self)	128	192	7.9	69	–	26.6
December 2021	Frequent urination, excessive perspiration and dryness of palate. Stressful mental status	Not on medication(self-dropped)	321	396	13.5	68	99	26.2
January 2022	Asymptomatic stage. Bowel –clear and mental status-occasional stressful episodes	On Ayurveda diet and Yoga	138	189	–	66	98	25.5
February 2022	Asymptomatic stage. Bowel-clear. Occasional stressful episodes	On Ayurveda diet and Yoga	129	146	–	64	98	24.7
March 2022	Asymptomatic stage. Bowel-clear. Occasional stressful episodes	On Ayurveda diet Yoga	97	101	6	61	97.6	23.5
May 2022	Asymptomatic stage. Bowel-clear. No stressful episodes	On Ayurveda diet Yoga	108	126	–	59	96.2	22.8
June 2022	Asymptomatic stage. Comfortable, good energy level	On Ayurveda Diet Yoga	102	114	5.6	58	95	22.4
July 2022	Asymptomatic stage. Comfortable, good energy level	On Ayurveda Diet Yoga	98	123	–	56	94.8	21.6
September 2022	Asymptomatic stage	On Ayurveda Diet Yoga	106	115	5.7	55	94.4	21.2

**Table No.2:** Yoga intervention prescribed

SI. No	Yoga practice		Effects
1	Breathing Practices (5 min)	Rapid breathing	–
		Hand stretch breathing, Tiger stretch breathing	
2	Loosening practices (5 min)		–
	Standing	Padahastasana-Ardha chakrasana vyayama, Trikonasana vyayama, Kati parivartana vyayama (spinal twist)	
	Sitting	Chakki chalana	
		Bhunamanasana	
	Supine	Pawana muktasana	
	Prone	Dhanurasana swing	
3	Relaxation (5 min)	Instant relaxation technique	
4	Suryanamaskara (5 min)	12 steps	Decreases hip circumference, helpful in glycemic outcomes [11]
5	Asanas (10 min each)		
	Standing	Ardhakati chakrasana	Improves functioning of insulin receptor cells, helps to increase glucose uptake by muscles [12]
		Parivrtta trikonasana	
	Sitting	Vakrasana	
		Ardha matsyendrasana	
	Prone	Bhujangasana	
		Dhanurasana	
	Supine	Pawanmuktasana	
		Matsyasana	
6	Relaxation (10 min)	Deep relaxation technique	
7	Kriya	Kapala bhati	Abdominal pressure created during exhalation improves the efficiency of β-cells of the pancreas [12]
8	Pranayama (10 min)	Nadi shuddhi	Improves health related fitness, body fat percentage etc. [12]
		Bhramari pranayama	
		Om chanting	
9	Meditation (20 minutes)	Cyclic meditation	Has benefits in stress management [13]

Presenting a case of a female with poorly controlled T2DM (HbA1c-13.5), successfully reversed using the dietary principles of Indian traditional medicine (Ayurveda diet) and therapeutic yoga practices. The diet comprised time restricted food intake, major meals of low glycemic index millet dishes, herbal smoothies and more green based salads and sprouts. Yogic practice encompassed yogic postures, breathing, & meditative techniques practiced for 60 minutes a day.

The novel treatment method, which focused on lifestyle change and incorporated Ayurveda dietary principles and yogic treatment, successfully reversed T2DM without causing long term side effects while also promoting holistic health improvement.

## 2 Patient's information

A 31-year-old female homemaker visited to diabetic specialty OPD on December 3, 2021, with the complaints of excessive perspiration, more frequent urination, and dryness of the palate persisting for two years, aggravated in the last six months. Two years ago, elevated blood glucose levels were detected during a routine test and diagnosed with T2DM, and physician had prescribed metformin with a dose of 1000mg per day. With this medication intake, she experienced bloating, diarrheal episode, and hypoglycemic symptoms. This led to non-compliance with the medication intake. She had a family history of T2DM. She is non-hypertensive, has no history of any systemic illness.

## 3 Clinical findings

The patient was presented with frequent urination, excessive perspiration, and dryness of palate for 2 years, which had worsened over the last 6 months. Diabetic medication adherence had been inconsistent for 2 years and completely stopped for the last 6 months. The patient expressed feelings of loneliness, irritability, anger issues, and a general disinterest in life, indicating stress. Additionally, she had a history of primary infertility after 6 years of marriage. Her menstrual history revealed menarche at 14 years, irregular menstruation for the past 8 years (with a cycle of 45–50 days), and scanty bleeding. The patient weighed 68 kg, with a BMI of 26 kg/m^2^ and a waist circumference of 99 cm. Her body constitution was vatapitta prakrati.

## 4 Timeline

The of treatment modalities and measured health parameters at each follow up are given in Table no. 1.

## 5 Diagnostic focus and assessment

**Fig. 1:**
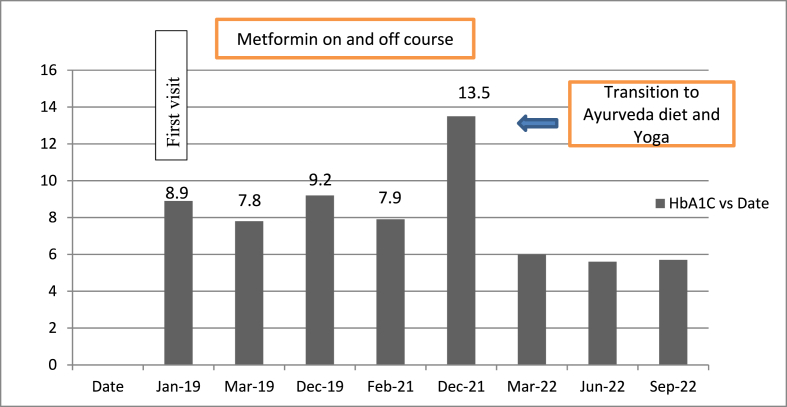
HbA1c prior and During Ayurveda Diet and Yoga.

On her visit, an initial evaluation of glycemic parameters [FBS: 321 mg/dl, PPBS: 396 mg/dl, HbA1C: 13.5] revealed inadequately controlled T2DM. Lipid profiles and the insulin resistance parameter (HOMA IR) were checked at the beginning. Prior treatment-measured health parameters during metformin on and off course and during treatment parameters were summarized [Table No. 1]. HbA1C decline throughout the course of the Ayurveda diet and yoga depicted in Fig. 1.

## 6 Therapeutic intervention

After a thorough analysis of the patient's history, we advised a plan to reverse her diabetic profile using ayurvedic principles of food and integrating a yoga protocol. Building upon our experience of witnessing reversal in many T2DM cases with this approach, we planned the same here with further refined methodology according to patient's body type, cultural beliefs, and acceptance.

The intervention strategy involved a personalized diet plan based on ayurvedic principles and therapeutic yoga. Motivation was provided through success stories and videos, along with handouts of recipes. Daily reminder messages were sent to enhance adherence. The patient attended weekly follow-ups at a diabetes specialty clinic for the first month, followed by monthly follow-ups for 9 months to track sustained improvement. The patient's symptoms were reduced after the initial month. The diet plan was based on Ayurvedic principles, incorporating nutrient-dense whole foods like millets and barley, soaked and sprouted beans and lentils, and healthy fats. It also includeed ayurvedic drinks, plant juices, and smoothies to support the reversal diet goals. Daily yoga sessions, conducted over 15 days by a certified instructor at our center, were complemented by one-hour nutritional counseling.

In therapeutic fasting, the patient was instructed to eat two major meals between 11 a.m. and 7 p.m. (upavasa-16-h fasting method). In the fasting period, any one of the following Ayurveda drinks was suggested: i.e, ash gourd juice, 1–2 glasses of copper-infused warm water, lemon water with apple cider vinegar, green tea (in moderation), or herbal-infused boiling water [added with Ayurveda herb powder, i.e. *Yashtimadhu* (*Glycyrrhiza glabra* Linn.), *Twak (Cinnamomum zeylanicum* Breyn Pennel), *Ela (Elatteria cardamom* maton), and *Dhanyaka* (*Coriandrum sativum*) filtered and used]. Two meals included low carbohydrate, medium protein, and healthy natural fat. We kept patient under Indian millet diet which included bajra (pearl millet), yava (barley), and diverse varieties of different millets, which made up 30% of her total meal using different preparations. We had incorporated 30–40% of the raw diet of herbal smoothies, chutneys, and salads. We primarily used pulses in the sprouted method. The first meal started with an herbal smoothie and a bowl of salad, followed by a millet lunch. Lunch had some good fat sources like ghee, coconut, flax, or peanuts in the form of chutney and nuts and seeds in salads. The patient was suggested to take herbal buttermilk at lunch. Dinner options predominantly contained soup made of moong, millet, barley, or quinoa, along with millet khichdi and kadhi (yogurt-based gravy). Whenever consuming food, she was advised to eat in a way that ensures fullness, wholesomeness, and satisfaction, aiming to keep the patient satiated for three to 4 h. If hunger strikes between meals, snacks based on lentils, pulses, and beans (e.g., sprout chat, fritters, peanut chat, ghee-roasted makhana) were recommended. These options are filling and do not cause a spike in insulin levels. The dietary intervention was designed to be palatable, easily accessible, and provide long-term sustainability for the patient. *[Annexure-I]*

A patient underwent 15 days of diabetic yoga training, supervised by a certified instructor. The program involved body-loosening practices, surya namaskara, yogic postures, and breathing techniques. The patient was encouraged to regularly report blood glucose levels, share food intake, and discuss challenges. Regular glucose checks and dietary modifications were made. Weight changes, BMI, and waist circumference measurements were also recorded.

This uniqueness of this strategy is combining therapeutic yoga with ayurvedic food concepts to treat T2DM. Ayurvedic treatment is tailored according to each person's unique body type and health status, with an eating plan based on acceptance, body type, and cultural values. The intervention includes various herbal drinks, herbal supplements, and upavasa, a novel method of purification and regeneration. Ayurveda emphasizes bhojana kala (meal frequency) and therapeutic fasting, which supports metabolic activity. Ayurvedic principles recommend foods high in barley, millet, greens, and good fat from seeds for diabetics. A therapeutic yoga program helps improve dosha balance and targets in managing diabetes.

## 7 Outcome and follow up

The following outcome measures obtained in this case: Serum HbA1C level (%), Fasting and Post prandial blood glucose level (mg/dl), Triglyceride level (mg/dl), Weight (kg), Waist circumference (cm) and BMI (kg/m^2^) Homa IR & Symptomatology.

The major outcome of this case is a significant reduction in HbA1c, shifting from inadequately controlled type 2 diabetes to near-normal levels. Noticeable reductions in fasting and postprandial blood glucose were observed. Weight, BMI, and waist circumference underwent marked changes, influenced greatly by the diet and yoga practices (Table No.1). No symptomatic episodes of hypoglycemia were noted during the intervention. The Ayurveda dietary intervention and yoga practices significantly impacted plasma triglyceride levels, decreasing from 170 mg/dl to 80 mg/dl post-follow-up. Improvements in Homa IR indicate a substantial reduction in insulin resistance, decreasing from 9.44 to 1.87.

After one month of diet and yoga intervention, the patient reported the regularization of micturition frequency, perspiration, and thirst. The same protocol continued for the next two months. Three months later, monthly follow-ups showed stable fasting and postprandial blood glucose levels, maintained for the entire six-month follow-up. Slight dietary modifications were then introduced, involving 3–4 days of therapeutic fasting weekly. On other days, the patient consumed a low-carbohydrate breakfast (moong or chickpea dosa, millet upma, sprout salad, organic egg recipes), while lunch and dinner remained unchanged. Occasional inclusion of brown or unpolished rice, low-glycemic index fruits, and vegetables compensated for cravings. Strict avoidance of Maida, wheat, sweets, ice-cream, chocolates, and bakery items was emphasized. Monthly examinations and lab recordings confirm the patient's comfort, good energy levels, and ability to perform household chores.

## 8 Discussion

The aim of diabetic reversal includes treating the root cause through lifestyle modifications. The present case report showed that the Ayurveda diet and yoga protocol significantly reduced diabetic symptoms and normalized poorly controlled glycemic parameters.

In Ayurveda, therapeutic fasting is a prime treatment modality in various diseases like obesity, skin disorders, diabetes, urinary disorders, and digestive disorders etc. Fasting told in Ayurveda acts by normalizing impaired Agni (digestive fire). It can be understood as how decreased consumption helps to utilize glycogen as first, then fat will be used for energy. Studies have shown that intermittent fasting decreases blood inflammatory markers and improve glucose regulation [14].

India has numerous nutritious millet varieties, rich in fiber with anti-inflammatory and antioxidant properties [15]. Ayurveda endorses versatile use of pulses and nuts in dosa, idli, snacks, cooked with herbal curries, made into salads, and chats. The diet incorporates ayurvedic herbs like cinnamon and liquorice as they boost metabolism and glucose and lipid control [16].

Yoga practices were incorporated into the patient's routine. Suryanamaskara and standing asanas rejuvenate pancreatic cells through alternating abdominal contraction and relaxation [12]. Yoga positively affects glucose utilization and fat redistribution in T2DM [17]. The patient experienced stress relief and reduced anxiety, likely due to yoga promoting tranquility, relaxation, and balanced energy [18]. The combined diet and yoga approach may work through insulin sensitization, anti-inflammatory, and antioxidant actions, with added benefits in stress reduction. This holistic approach also contributed to sustaining the reversal of diabetes, evidenced by stable glycemic parameters during the follow-up period. Overall, this integrated approach provides a comprehensive and effective diabetes management plan, emphasizing dietary changes, therapeutic fasting, physical exercise, and mindfulness practices based on Ayurvedic principles.

## 9 Patient perspective

The patient is symptom-free from T2DM, experiencing heightened energy levels with reduced cravings and hunger. Satisfied with healthy weight loss, she is accustomed to the diet and yoga protocol, leading to an improved quality of life and effective lifestyle management.

Informed consent for publication was obtained form the patient.

## 10 Conclusion

This case demonstrates the effectiveness of the ayurveda diet and yoga practices in reversing insulin resistance and maintaining glycemic parameters. Additionally, the patient lost around 13 kg of body weight, experienced a reduction in waist circumference, and lowered serum triglyceride levels. The patient was able to maintain a normal diabetic profile without the need for pharmacotherapy intervention.

## Source of funding

Nil

Author Contributions

SK: Conceptualization, Methodology, Investigation, Resources, Writing - Original draft preparation, Writing - Reviewing and Editing, visualization, supervision, project administration. BRT: Methodology, Investigation, Supervision, Data curation, Writing - Reviewing and Editing. RP: Methodology, Investigation ,Supervision, Data curation, Writing - Reviewing and Editing. LSD: Methodology, Visualization, Writing - Reviewing and Editing, Data curation.

## Declaration of generative AI and AI-assisted technologies in the writing process

During the preparation of this work the authors used chat GPT 3.5 AI assistance to grammar and readability. After using this tool/service, the authors reviewed and edited the content as needed and take full responsibility for the content of the publication.

## Conflict of interest

None declared.

## Appendix A Supplementary data

Menu plan and diet chart Menu plan and diet chart
