# Supplementary material for: Reversal of type 2 diabetes mellitus through integrated Ayurveda dietary protocol – A case report
Source: J Ayurveda Integr Med. 2024 Jul 23;15(4):100946. doi: 10.1016/j.jaim.2024.100946 (PMC11321381; doi:10.1016/j.jaim.2024.100946)
Supplement: Menu plan and diet chart [file mmc1.pdf]

ANNEXURE-1

Diet chart (menu plan)

**Timing**<br>**Food Diary**	**Menu(with serving quantity)**
**Ayurveda drink**<br>**B/w 7 to 8 am**<br>**(Any One)**	1. Ash gourd juice- 150 ml (1 glass)
	2. Copper charged water -1 to 2 Glass
	3. Lime water(added with 1tsf Apple cider vinegar and a pinch of pink salt)-150 ml
	4. Herbal infused drink(muleti, cinnamon, cardamom & coriander)-150 ml
	5. Green tea- 1 cup
**First meal**<br>**11am**	• Herbal smoothie-100 ml<br>• Mix veg Salad*-1 bowl/ moong sprout-1 bowl/veg stir fry-1 bowl(with grated coconut ,nuts and seeds toppins)<br>*****Ideally 50% of the plate is raw/salad<br>• Roti(made of Bajra/pearl barley/Sorghum)- 1 to 2 small size with cow's ghee<br>• Vegetable sabzi<br>• Moong dal/sambar-1 bowl<br>• Peanut ⁄Flax seed /fenugreek seed chutney-10 grams<br>• Herbal buttermilk-200 ml
**Mid-Evening healthy snack(optional-only if hungry)**<br>***Any one***<br>**5 pm**	1. Almond -4<br>+ Roasted or soaked Peanuts-1 handful<br>+Walnut-2
	2. Ghee Roasted makhanas added with Rock salt+ Turmeric -1 cup
	3. Roasted Pea nut chat-1 handful
	4. Whole moong or chana dal fritters/Bindi fry/Brinjal tawa fry/ Methi Muthia/sprout chat -1 plate (Any one)
**Dinner**<br>**(7 pm)**	• Herbal Soup-1 bowl(add millet/pearl barley/moong)<br>• Millet kichdi -1 plate(added with ghee)<br>• Herbal Kadhi / raita-100ml
**General tips**	• Drink sufficient water<br>• No fruits,maida,wheat,dairy,sweets,ice creams and chocolates<br>• Regular yoga(60 minutes daily)<br>• Detox water sip by sip<br>o Option-1(lime water -sip by sip)<br>o Option -2: Half tsf twice daily in a glass of water(dry roasted powder of Cinnamon,methi,carom,cloves,dry ginger,cardamom,fennel)

<u>Exchange List/Day</u>

[Protein: 45 gm/day (+ or – 5 g), CHO: 195 gm, Fat: 26.5gm/day]

Menu	Exchange	Gram	Calorie	Protein	CHO	Fat
**Cereals**	3	90	300	9	54	2.1
**Rice**	2	60	200	5	46	0.4
**Pulses**	2	60	180	13	30	1
**Buttermilk**	1	100 ml	26	2	2	1
**Curd**	1	100	62	4	4	3
**Vegetable**	4	400	160	8	26	1.7
**Fruits**	1	50-75	50	1	10	-
**Oils and Fats**	3	15	135	-	-	15
**Nuts and oilseeds**	1	20	100	3.5	5	7.5
**Total**	**-**	**-**	**1213 kcal**	**45.5 gm**	**177 gm**	**31.7 gm**

Recipe Book

**Smoothie Recipes**			
**1.Dandelian smoothie**<br>Ingredients;<br>Dandelion leaves-1 handful<br>Cucumber-1<br>Green apple-1<br>Lime juice-2 tsf<br>Procedure: Grind the ingredients (except lemon).Filter and add lime juice. Drink 1 glass per day	**2.Carrot Smoothie**<br>**Ingredients**<br>1 carrot<br>+1 Tomato<br>+1 green apple<br>+Ginger –small piece<br>+1/2 cup grated coconut<br>Mix well in blender<br>Drink 1 glass per day	**3.Herbs mix smoothie**<br>Coriander-10 leaves<br>+ Betel -1 leaf<br>+ Basil-4 leaves<br>+ mint-4 leaves<br>+ Curry leaves-5-6 leaves<br>+1 green apple<br>+ 1 glass water<br>+ Cinnamon -1/2 stick small pieces<br>+ Black pepper-2<br>+ Turmeric-1/2 tsf<br>+ Rock salt<br>Mix well in blender, consume fresh 1 to 2 glasses per day	**4.Bottle gourd smoothie**<br>1 Medium size Bottle gourd<br>+1 green apple<br>+1 Tomato<br>Mix well in blender<br>Consume 1 glass per day

Herbal Raita recipes

<table>
<tr><th>Giloy buttermilk</th><th>Avla buttermilk</th><th>Curry leaves buttermilk</th><th>Fenugreek seed buttermilk</th></tr>
<tr>
<td>Ingredients
Coriander leaves-1/2 cup, Green chilli - 2,Black pepper- 6 to 8,Jeer- 1 tsf, Giloy leave -½ cup, Grated coconut - 1 cup, Garlic- 4 cloves, Udad dal-2tsf,Curd -1 cup, Mustard- 1tsf ,ghee- 2 tsf, Salt as per taste
Procedure: In a pan, add 2 tsf of ghee,cumin seeds, black pepper, curry leaves and fry well, Then add green chilli, giloy leaves and fry a little.(don't fry much as potency of giloy leaves should not go). Add a cup of grated coconut.

After cooling, shift it to the jar& grind well. Transfer it to a bowl. Add salt as per taste and later add a cup of organic curd. Mix well .Add a tempering(by adding ghee, garlic, mustard , udad dal and curry leaves).Serve with Kichdi or millet rice</td>
<td>Ingredients
Avla -Deseeded -2 to 3,Jeera-1/2 tsf,Pepper-8 to 9,Grated coconut-1 cup, salt as per taste, Ghee ½ tsf,1 cup curd
Procedure: In a mixer grinder, take a cup of coconut, slightly fried jeera and pepper, avla pieces and grind to smooth paste. Add this fine paste to curd and mix well .See the consistency. Add the tempering with ghee. Serve with Millet rice or Kichdi</td>
<td>Ingredients
1 cup washed curry leaves,1 cup curd,1 cup grated coconut,2 tsf water,½ tsf jeera, cumin seeds,4 to 5 black pepper,1 tsf ghee,½ tsf salt,½ tsf mustard seeds,1/ 2tsf jeera,1 red chilli broken into pieces
Procedure: Take a pan, add a tsf of ghee. Add pepper, cumin, curry leaves. Fry for 4 minutes. Add coconut and slightly warm. After cooling, add to grinder, add salt, little water and grind coarsely. After coarse grinding, add curd and again grind a little. For tempering, in a small pan take 1 tsf of ghee. Add mustard, jeera and a chilli. Fry till mustard sputters. Add it to the grinded mixture. Mix thoroughly and serve with millet rice or millet kichdi</td>
<td>Ingredients
Oil- 1tsf,fenugreek seeds 2 tsf,Red chilli 2 ,Coconut 1 cup ,Curd 1 cup
Procedure: In a pan, add a tablespoon of oil or ghee. Add 2 tsf fenugreek seeds, add a red chilli. Sauté for a minute. Transfer to a mixture. Add a cup of coconut, salt and little water. Grind for a course mixture. Transfer to the content. Add 1 cup buttermilk. Mix well. For a tempering, take a pan, add ghee, mustard, curry leaves, udad dal, red chillies .sauté well and add to the grind mixture. Serve it with kichdi or millet rice</td>
</tr>
</table>

Soup Recipes			
**Moong soup**	**Millet soup**	**Barley soup**	**Basil vegetable soup**
**Ingredients:**<br>•Green gram - 1/2 cup<br>•water - 3 to 4 cup • black pepper powder - 1/2 tsp<br>•Ghee-1 tsf,garlic-2 cloves, salt as per taste<br>**Procedure**<br>Take green gram, soak overnight, Add 4 cups of water and boil till cooked. Strain the water. Add salt, pepper and mix thoroughly. Take a pan and heat, Add 2 tsf ghee, mustard seeds and crushed garlic. Pour it on the soup. Serve hot	**Ingredients**<br>Foxtail millet-3tsf(soaked for 3hrs)<br>Ginger-1/2 piece<br>Ghee-2 tsf<br>Pepper-2 tsf<br>Vegetable-Palak chopped-2 leaves ,Carrot chopped-1<br>Green peas-1/4th bowl<br>**Procedure**<br>Take a pan, into this add ghee and add chopped ginger, sauté for 2 minutes, next will be adding vegetables, fry a little. Add a pinch of pink salt, mix well and cover with lid .cook for 2 minutes. Now add grind millet puree. Add 2 cups of water. Adjust soup consistency. Add pepper powder and cook for 5 minutes. Serve hot.	**Ingredients**<br><br>Barley-1/2 cup,Water-3 cups, Finely chopped carrot-2 tsp, finely chopped garlic-1 tsf, Finely chopped ginger-1 inch, Salt to taste, white pepper powder-1/2 tsf,oil-1 tsf,<br><br>**Procedure**<br><br>Take barley in a bowl, Soak for 4 hours, Cover and Cook for 3 whistles, Let the pressure release naturally, Strain it, Add 2 tsf of cooked barley in a mixer grinder, Grind to smooth, Add a tsf of oil in a pan.Add ginger and garlic and saute them for a minute, Add sweet corn,chopped carrot and peas, Saute them for 2 to 3 minutes, Add 2 to 3 tsp of cooked barley. Saute them for a minute, Add barley water ,1/2 to 1 cup of water and salt, Add salt,Barley paste and mix well, Cover and cook for 6-8 minutes, Add pepper powder. Serve hot	**Ingredients**<br><br>½ cup finely chopped onions,¾ cup chopped capsicum,¼ cup finely chopped French beans,¼ cup chopped carrot ,¼ cup chopped cabbage,2 tbsp finely chopped celery,¼ cup fresh basil leaves ,2 tsp olive oil,1 tsp finely chopped garlic,1 tsf finely chopped green chilli ,4 cup vegetable stock ,Salt to taste<br><br>Procedure<br><br>Heat olive oil in a pan.Add garlic,green chillies,saute for few minutes. Add carrot,cabbage,French beans, green chillies, capsicum.Saute for 4 to 5 minute. Add vegetable stock,boil for 4 to 5 minutes. Add Basil leaves,pepper powder and serve hot.
